# Variation in susceptibility of African *Plasmodium falciparum* malaria parasites to TEP1 mediated killing in *Anopheles gambiae* mosquitoes

**DOI:** 10.1038/srep20440

**Published:** 2016-02-10

**Authors:** Maarten Eldering, Isabelle Morlais, Geert-Jan van Gemert, Marga van de Vegte-Bolmer, Wouter Graumans, Rianne Siebelink-Stoter, Martijn Vos, Luc Abate, Will Roeffen, Teun Bousema, Elena A. Levashina, Robert W. Sauerwein

**Affiliations:** 1 Department of Medical Microbiology, Radboud University Medical Centre, Nijmegen, The Netherlands; 2 Vector Biology Unit, Max Planck Institute for Infection Biology, Berlin, Germany; 3 UMR MIVEGEC UM1-CNRS 5290-IRD 224, Institut de Recherche pour le Développement, Montpellier cedex, France; 4 Department of Immunology and Infection, London School of Hygiene and Tropical Medicine, London, United Kingdom

## Abstract

*Anopheles gambiae* s.s. mosquitoes are efficient vectors for *Plasmodium falciparum*, although variation exists in their susceptibility to infection. This variation depends partly on the thioester-containing protein 1 (TEP1) and TEP depletion results in significantly elevated numbers of oocysts in susceptible and resistant mosquitoes. Polymorphism in the *Plasmodium* gene coding for the surface protein Pfs47 modulates resistance of some parasite laboratory strains to TEP1-mediated killing. Here, we examined resistance of *P. falciparum* isolates of African origin (NF54, NF165 and NF166) to TEP1-mediated killing in a susceptible Ngousso and a refractory L3–5 strain of *A. gambiae*. All parasite clones successfully developed in susceptible mosquitoes with limited evidence for an impact of TEP1 on transmission efficiency. In contrast, NF166 and NF165 oocyst densities were strongly reduced in refractory mosquitoes and *TEP1* silencing significantly increased oocyst densities. Our results reveal differences between African *P. falciparum* strains in their capacity to evade TEP1-mediated killing in resistant mosquitoes. There was no significant correlation between *Pfs47* genotype and resistance of a given *P. falciparum* isolate for TEP1 killing. These data suggest that polymorphisms in this locus are not the sole mediators of immune evasion of African malaria parasites.

Malaria is one of the most important infectious diseases worldwide with an estimated 198 million cases and 584,000 deaths annually^1^. The responsible *Plasmodium* protozoan parasites undergo a complex sporogonic life cycle once ingested by female *Anopheles* mosquitoes from an infected human host. Male and female gametocytes are taken up and fuse to form a motile ookinete. The ookinete then penetrates the mosquito midgut epithelium to establish infection in the basal labyrinth where it is exposed to soluble immune factors secreted by mosquito blood cells. Surviving ookinetes settle under the basal lamina to differentiate into an oocyst that matures over time and eventually ruptures to release thousands of sporozoites that invade the salivary glands and render the mosquito infectious for humans^2^.

*A. gambiae* from the field^3^,^4^ or the lab^5^,^6^ show variable susceptibility to *Plasmodium* parasites, which may be partly attributed to the efficiency of mosquito immune factors to kill ookinetes^7^,^8^,^9^. The immune response is mediated by a series of genes whose expression is induced by such stimuli as blood feeding, infection with bacteria and/or *Plasmodium* parasites and sterile wounding^10^.

REL1^11^,^12^,^13^ and REL2^14^,^15^,^16^ together with Jak/Stat^17^ and JNK^18^,^19^ are the four major mosquito immune signaling pathways. Thioester-containing protein 1 (TEP1) is regulated by REL1, REL2 and JNK pathways, reflecting its central importance in mosquito immune responses^10^. TEP1 is secreted by hemocytes into the hemolymph and its activity is controlled by a complex consisting of two leucine-rich repeat (LRR) proteins, LRIM and APL1C. The LRR complex maintains circulation of the activated form of TEP1 in the hemolymph^15^,^20^. Binding of TEP1 to the surface of invading ookinetes initiates near total lysis of the concomitant parasite population^21^. Knockdown of *TEP1* in the *A. gambiae* - *Plasmodium berghei* laboratory model results in a 3- to 5-fold increase in oocyst numbers in susceptible and resistant mosquitoes^21^,^22^. Depending on the parasite genetic composition, TEP1 also mediates *P. falciparum* ookinete killing in *A. gambiae* mosquitoes^7^.

The refractory *A. gambiae* L3–5 (or R) strain was initially selected from the susceptible G3 (S) strain for its high resistance phenotype for several *Plasmodium* species^5^,^23^. In the L3–5 strain, the majority of tested parasite species are killed and melanized within the first two days after infectious blood feeding. Interestingly, silencing of the members of the complement-like system, including TEP1, LRRs and NOX5/HPX2, renders these resistant mosquitoes fully susceptible to infections with *P. berghei*, demonstrating the key role in this defense mechanism. Moreover, polymorphism at the *TEP1* locus is directly responsible for the differences between R and S strains in *P. berghei* killing; R strain is homozygous for TEP1*R1 allele, whereas S strains contain TEP1*S1/S2/R2 alleles^24^. Although all alleles confer variable degrees of resistance to malaria parasites, TEP1*R1 confers the highest levels of resistance^22^.

Recent reports revealed differences in sporogonic development between African (NF54, GB4) and Brazilian (7 G8) *P. falciparum* laboratory strains in R mosquitoes, where NF54 was resistant to TEP1 mediated killing, while 7 G8 was highly susceptible^7^,^25^. Based on these results, it was suggested that sympatric African parasites may have developed means to evade TEP1 killing^25^. Interestingly, resistance of the parasites to TEP1 correlated with the polymorphism in the *Pfs47* gene encoding a cysteine-rich gametocyte surface protein. In *A. gambiae*, infections with the *Pfs47KO* strain were completely aborted by the mosquito complement-like system^26^, suggesting that both mosquito and parasite genetic factors contribute to the outcome of infections. An elegant evolutionary hypothesis was put forward suggesting that polymorphism at the *Pfs47* locus permitted adaptation of African parasites to the mosquito complement-like system^26^. These conclusions, however, were based on studies with a single laboratory NF54 strain of likely African origin that has been maintained in the culture for more than 30 years.

Here we examined whether variation at the *Pfs47* locus correlates with the sensitivity of different African *P. falciparum* strains to TEP1 mediated killing. We report that two new *P. falciparum* strains NF165 (originating from Malawi) and NF166 (originating from Guinea) differ in their resistance to TEP1-mediated killing. Genotyping *Pfs47* in a series of African *P. falciparum* parasites demonstrate that variability in *Pfs47* does not correlate with resistance to TEP1-dependent ookinete killing. Sequence comparison revealed striking divergence between *Pfs47* genotype in NF54 (and its relative 3D7) and other African *P. falciparum* isolates, suggesting that currently circulating *P. falciparum* isolates may be more susceptible to TEP1-mediated killing than initially thought.

## Results

### Resistance of *P. falciparum* strains to TEP1-mediated killing in susceptible *A. gambiae*

We first examined the susceptibility of the recently colonized Ngousso mosquitoes to *P. falciparum* NF54 strain and two freshly isolated strains: NF165 (Malawi) and NF166 (Guinea). Ngousso strain is a mix of TEP1*S alleles (0,7 - *S1/S1; 0,2 - *S1/S2; 0,1 - *S2/S2) and has been widely used for experimental infections in Cameroon^27^. Young females were injected with dsRNA to silence expression of *TEP1*, whereas noninjected mosquitoes and mosquitoes injected with dsRNA of an unrelated gene (*dsLacZ*) served as controls. Efficiency of *TEP1* silencing was evaluated by immunoblotting of the hemolymph extracts collected from *dsLacZ* and *dsTEP1* mosquitoes four days after injection (Fig. S1). Two experiments with *P. berghei* infected mice confirmed that *TEP1* silencing was efficient as it caused a significant increase in oocyst numbers in the *dsTEP1* as compared to *dsLacZ* mosquitoes (Fig. S2).

**Figure 1:**
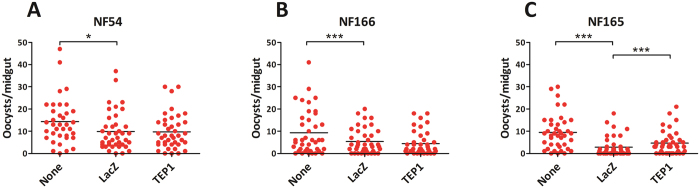
Effect of *TEP1* silencing and wounding in *A. gambiae* S mosquitoes on *P. falciparum* (strain NF54, NF166 and NF165) infection. Female mosquitoes were injected with *dsLacZ* (control) or *dsTEP1* 4 days prior to feeding on a *P. falciparum* gametocyte mixture. Oocysts were visualized on day 7–8 post infection (p.i.) by mercurochrome staining of midguts and counted by microscopy. (**A**) Effect of wounding and *TEP1* silencing for *P. falciparum* NF54. (**B**) Effect of wounding and TEP1 silencing for *P. falciparum* NF166. (**C**) Effect of wounding and *TEP1* silencing for *P. falciparum* NF165. Data shown of individual experiments, for all strains phenotypes were confirmed in at least three independent experiments. Statistical analysis was done on pooled experiments and significance between groups are indicated: *p < 0.05, **p < 0.01, ***p < 0.001 (negative binomial regression model).

In parallel, injected mosquitoes were fed on a membrane feeder system containing *P. falciparum* gametocyte culture. At least three independent infections were performed with *P. falciparum* strains NF54, NF166 and NF165. We compared oocyst numbers in noninjected and injected mosquitoes and observed a significant effect of wounding on infections with all three tested *P. falciparum* strains (Table S1 and Fig. 1A–C). In line with the previous reports for NF54, no increase in oocyst numbers was observed in *dsTEP1* as compared to control *dsLacZ* mosquitoes (p = 0.30; Table 1 and Fig. 1A). Similarly, *TEP1* silencing did not affect development of NF166 (p = 0.25, Table 1 and Fig. 1B). A statistically significant 1.7-fold increase in oocyst burden after *TEP1* knockdown was detected for NF165 (p < 0.001, 95% CI 1.32–2.19, Table 1 and Fig. 1C). These results suggest that *P. falciparum* clones of African origin differ in their susceptibility to TEP1-mediated killing in a susceptible *A. gambiae* strain.

### Resistance of *P. falciparum* strains to TEP1-mediated killing in resistant *A. gambiae*

We determined the degree of resistance of the L3–5 mosquitoes to infection with *P. falciparum* isolates and assessed the contribution of the complement-like factor TEP1 to this resistance. To this end, *dsLacZ* and *dsTEP1* were injected into the thorax of young R females, whereas noninjected mosquitoes served as a control. Four days after injection, hemolymph of *dsLacZ* and *dsTEP1* injected mosquitoes was collected and immunoblotting analysis was performed to evaluate efficiency of *TEP1* silencing at the protein level (Fig. S3). As described above, two independent infections with *P. berghei* parasites confirmed the functional efficiency of TEP1 depletion. Ookinete development was almost completely aborted in the *dsLacZ* group and melanized parasites were observed in the mosquito midguts. As expected, *TEP1* silencing significantly increased oocyst numbers and completely inhibited melanization. (Fig. S4).

**Figure 2:**
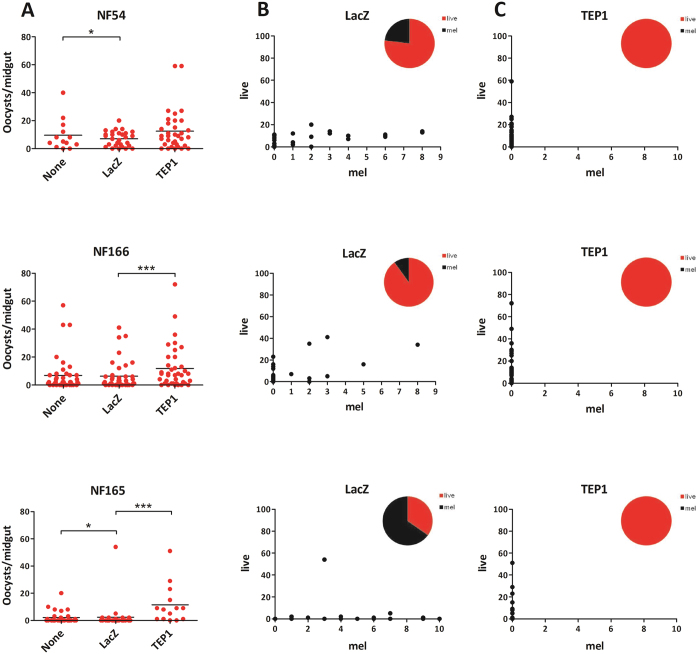
Effect of wounding and of *TEP1* silencing on development of *P. falciparum* (NF54, NF166 and NF165 439 strains) in *A. gambiae* R mosquitoes. Female mosquitoes were injected with *dsLacZ* (control) or *dsTEP1* 4 days 440 prior to feeding on a *P. falciparum* gametocyte mixture. Oocysts were visualized on day 7–8 p.i. by 441 mercurochrome staining of midguts and counted by microscopy. (**A**) Comparison of *P. falciparum* live oocyst 442 counts between control groups (none vs. *dsLacZ*) for effect of wounding and between *dsLacZ* injected and TEP1 443 silenced group. (**B**) Numbers of live and melanized oocyst on individual midguts in the control injected group. 444 Proportion of live/melanized is depicted in the pie chart. (**C**) Numbers of live and melanized oocysts on 445 individual midguts after *TEP1* silencing. Proportion of live/melanized is depicted in the pie chart. Data shown of 446 individual experiments, for all strains phenotypes were confirmed in at least three independent experiments. 447 Statistical analysis was done on pooled experiments and significance between groups are indicated: *p < 0.05, **p < 0.01, ***p < 0.001 (negative binomial regression model).

In parallel, injected mosquitoes were fed on a membrane feeder system containing *P. falciparum* gametocytes. We performed three independent infections of L3–5 mosquitoes with the *P. falciparum* isolates. Overall infection levels were statistically significant lower in L3–5 than in Ngousso. Wounding by microinjection had a statistically significant effect on parasite development for NF54 and NF165. This wounding effect was not observed for NF166 (Table S1 and Fig. 2A). We then compared the development of the three parasite clones in the presence and absence of TEP1. As reported previously, silencing of *TEP1* had no effect on NF54 survival and did not restore the lower infection burden resulting from wounding, suggesting a TEP1-independent mechanism of ookinete killing. In contrast to NF54, *TEP1* silencing in L3–5 mosquitoes significantly increased oocyst loads of two other strains: 3.6-fold for NF165 (95% CI 2.36–5.39, p < 0.001) and 1.8-fold for NF166 (95% CI 1.31–2.40, p < 0.001) (Table 1 and Fig. 2A). Strikingly, variable numbers of melanized ookinetes were detected in infections with all tested parasite strains. Melanized ookinetes were most commonly observed in NF165 infections (up to 66%), followed by in NF54 (12%) and NF166 (4%). *TEP1* silencing completely abolished ookinete melanization in all three strains, suggesting a key role of the complement-like molecule in this defense mechanism (Fig. 2B,C).

Taken together, these results suggest that African *P. falciparum* strains, including NF54, do not evade mosquito immune responses completely and suffer significant losses in terms of successful oocyst formation. Moreover, the efficiency of the mosquito immune responses depends on the genetic background of the mosquito and of the parasite. While all examined *Plasmodium* isolates suffer substantial losses in R mosquitoes, TEP1 mediates killing of NF165 and, to a lesser extent, NF166 isolates. As silencing of *TEP1* fails to rescue killing of NF54 parasites in R mosquitoes, we suggest that TEP1-resistant parasite isolates are eliminated by currently uncharacterized immune responses.

We conclude that African *P. falciparum* strains vary in their susceptibility to TEP1-mediated killing and that the outcome of infection depends on the combination of genetic backgrounds of both mosquitoes and parasites.

### *Pfs47* polymorphism in *P. falciparum* isolates from Africa

To examine whether *Pfs47* polymorphism determines the outcome of infections with natural *P. falciparum* isolates, we explored genetic diversity in the immune-variable region that was proposed to underlie complement susceptibility of African *P. falciparum* strains^26^. To this end, the region of interest of *Pfs47* was PCR-amplified and sequenced from the NF54, NF165 and NF166 strains and from field isolates from Cameroon^27^. In addition, we compared *Pfs47* sequences of laboratory and field *P. falciparum* isolates of diverse geographic origins available in the public databases (MalariaGen project, PlasmoDB). The *Pfs47* sequences of NF166 and NF165 displayed strong similarities with other African strains (S2 Table) but were markedly different from parasite strains from Latin America and South-East Asia and our reference *P. falciparum* strains (NF54 and 3D7). For example, *Pfs47* sequences of *P. falciparum* 7G8 (Latin America, highly susceptible) and NF135 (South-East Asia) displayed three unique SNPs compared to all strains originated from sub Saharan Africa (S2 Table).

Regardless of origin, *C581A*−>P194H polymorphism was fixed in all 26 *Pfs47* sequences, except for the reference strains NF54 and 3D7, and a mixed isolate MA7 from Cameroon, which was heterozygous for *C/A* (S2 Table). Interestingly, this was the only SNP that distinguished the most TEP1-sensitive *P. falciparum* strain identified in this study NF165 from the TEP1-evasive NF54. The NF166 strain was more divergent from NF54/3D7 and in addition to *C581A*, displayed three additional missense SNPs (*G657A*−>M219I; *A742T*−>I248L, *A814T*−>N272Y). Sequenced isolates from Cameroon were very similar to those of NF165 and NF166 showing no correlation between *Pfs47* genotype and resistance to TEP1-mediated killing. Indeed, six out of 10 potentially “susceptible” genotypes of NF165-type were resistant to TEP1 killing in S mosquitoes. Conversely, two potentially “resistant” NF166-type genotypes were susceptible to TEP1 (S2 Table).

As no significant correlation between resistance of a given *P. falciparum* isolate to TEP1 killing and *Pfs47* genotype was detected, we concluded that polymorphism at this locus alone does not account for variation in TEP1-mediated immune evasion of African malaria parasites. Our results further suggest that the laboratory 3D7 and NF54 strains represent an exception, rather than a rule in regard to common *Pfs47* variants, and cannot be considered to be representative of other *P. falciparum* isolates from Africa in this context.

## Discussion

Molecular mechanisms of mosquito immune responses to *P. falciparum* infection and of parasite immune-evasion strategies are important for our understanding of malaria transmission dynamics. It has been suggested that the high disease burden in Africa may be (partly) attributed to the parasite adaptation of African *P. falciparum* strains to their sympatric vector through evasion of TEP1-mediated killing^25^. We present evidence that parasite populations in sub Saharan Africa differ in their ability to effectively evade TEP1 effector functions and that these differences in susceptibility are most pronounced in the resistant strain of *A. gambiae*.

The reason for variation in susceptibility of *P. falciparum* isolates to TEP1-mediated killing is currently unknown. Although mosquito populations with the fixed TEP1*R1 resistant allele have been documented in *A. coluzzii*, formerly known as the *A. gambiae* M-form, from Mali and Burkina Faso, their resistance to *P. falciparum* infections has not been experimentally evaluated^28^,^29^. In areas where R mosquitoes are prevalent, parasites that developed ways to evade TEP1-mediated killing would have a considerable transmission advantage whilst the selective pressure would be less pronounced in areas with lower prevalence of R mosquitoes. Susceptibility of NF165 to TEP1 mediated killing reported here could thus result from a lower exposure to R mosquito populations. NF165 was isolated in Malawi in East Africa, where TEP1*R1 allele is rare, supporting this hypothesis. In contrast, NF166 originates from Guinea, that borders Mali where TEP1*R1 mosquito populations are more abundant. Similarly, no TEP1*R1 alleles have been described in Cameroon and parasite resistance to mosquito immune pressure may have developed through other, TEP1-independent, mechanisms. This is in line with the previous observations on TEP1 susceptibility of the Brazilian *P. falciparum* 7G8 which is transmitted by the Anopheline vectors that lack TEP1 driven parasite killing^25^. We therefore propose that evolution of parasite resistance to TEP1 killing may result in adaptations within mosquito populations that result in variation at global (South America as opposed to Africa) and more local scale. Our findings indicate that resistance to *P. falciparum* in R mosquitoes is only partially determined by TEP1 as removal of this protein does not fully restore oocyst densities to levels seen in S mosquitoes.

We observed a considerable negative effect of wounding on oocyst development for all *P. falciparum* strains in the S mosquitoes and for two out of three *P. falciparum* strains in the R mosquitoes. Previous studies in S mosquitoes have shown that experimentally-induced wounding promotes *P. falciparum* killing via REL1/REL2 and AP-1/Fos pathways that control expression of *TEP1* and a gene encoding transglutaminase 2, respectively^18^,^27^. As injection caused a significant reduction in the survival of TEP1-resistant NF54 parasites, we conclude that the observed wounding phenotype does not require TEP1 function. In fact, the wounding effect was less pronounced in R compared to S mosquitoes. The wounding thereby does not affect our conclusions based on the results obtained with TEP1 depletion by micro-injection.

While questions remain about the exact mechanism of TEP1-mediated killing in *A. gambiae*, our data demonstrate that TEP1 is an important determinant of the success of *P. falciparum* transmission. Our findings suggest that African *P. falciparum* populations vary in their susceptibility to TEP1-mediated killing, and that this variation is more complex and goes beyond the *Pfs47* locus.

## Materials & Methods

### Mosquito rearing, parasite infections and mosquito midgut dissections

*A. gambiae* Ngousso (S) and *A. gambiae* L3–5 (R) mosquitoes were reared at 27–30 ^o^C and 70–80% humidity in a 12/12 hour day/night cycle. For infection experiments, mosquitoes were fed on a glass membrane feeder system containing ~1,25 mL of *P. falciparum* culture mixture as described before^30^. Three *P. falciparum* strains, NF54 (African origin)^31^, NF165 (Malawi) and NF166 (Guinea), were cultured as described before^32^. Female BALB⁄cByJ and C57BL ⁄6J, 8 weeks of age, were purchased from Elevage Janvier (Le Genest Saint Isle, France). Rodent experiments were performed according to the regulations of the Dutch “Animal On Experimentation act” and the European guidelines 86 ⁄ 609 ⁄EEG. Approval was obtained from the Radboud University Experimental Animal Ethical Committee (RUDEC 2009-019, RUDEC 2009-225). *P. berghei* (ANKA) was passaged in C57BL/6J mice and parasitemia was assessed using Giemsa-stained slides of tail blood. Animals were anesthetized using the isoflurane-anaesthesia system and mosquitoes were fed for 10 min.

Unfed mosquitoes were removed from the samples. Blood-fed mosquitoes were maintained at 26 °C or 21 °C, for *P. falciparum* and *P. berghei* infections, respectively, and 70–80% humidity. Midguts were dissected 6–9 days post infection in 1% merchurochrome staining solution. Numbers of oocyst were counted using an Axio Scope A1 microscope (Zeiss).

### Polymorphism detection by PCR

Genomic DNA was isolated from mosquitoes and used as a template for PCR genotyping. Specific primers: *TEP1* fwd 5′-*AAAGCTACGAATTTGTTGCGTCA*-3′ as a universal primer, *TEP1-S* rev 5′-*ATAGTTCATTCCGTTTTGGATTACCA*-3′ for susceptible strain and *TEP1-R* rev 5′-*CCTCTGCGTGCTTTGCTT*-3′ for resistant strain, amplified fragments of 372 bp and 349 bp, respectively. Standard program was used; 4 min at 94 °C, then 30 s at 94 °C, 30 s at 54 °C, 60 s at 72 °C for 35 cycles, and finally 7 min at 72 °C.

### dsRNA synthesis, injection and immunoblotting

Double-stranded RNAs were synthesized from plasmids pLL17 (*dsTEP1*) and pLL100 (*dsLacZ*) using the T7 Megascript Kit (Ambion) as described previously^10^,^21^. One-day-old female mosquitoes were anesthetized on a CO_2_ pad, injected with 69 nL of dsRNA (3 μg/μL) using a Nanoject II injector system (Drummond; Broomall, PA) and 4–8 days later fed with the *P. falciparum* cultures or on infected *P. berghei* mice. Unfed mosquitoes were removed from cages and the efficiency of gene silencing was monitored by immunoblotting. Hemolymph of 7–8 dsRNA treated mosquitoes was collected by proboscis clipping into denaturizing protein loading buffer (1:2 SDS, 1:10 DTT in PBS), and samples were separated by 7% SDS-PAGE. After protein membrane transfer, the specificity of *TEP1* knockdown was examined using TEP1-specific antibodies (1:500) revealing 165 kDa full-length and 80 kDa cleaved forms of the protein. Antibodies against prophenoloxidase 2 (PPO2) (1:10,000) were used as a loading control. Bound antibodies were detected by anti-rabbit IgG conjugated to HRP at 1:10,000 and addition of chromogen and peroxide.

### *Pfs47* DNA sequence analysis

Genomic DNA of *P. falciparum* strains NF54, NF165 and NF166 was isolated from asexual parasite culture material using QIAmp DNA Blood mini kit (Qiagen, NL). Procedures for recovery of DNA from *P. falciparum* African isolates were described in Nsango *et al.* 2012. Briefly, gametocytes were purified by magnetic separation of 1 mL of serum-free blood on a LD separation column using the MACS system (Miltenyi Biotec, Germany) and DNA was extracted with DNAzol according to the manufacturer’s instructions (Molecular Research Center, USA).

Specific primers were used; *BVS300* fwd: ‘5-*TACATTCAAATAACTCAGAGGGTAAC*-3′ and *BVS301* rev: ‘5-*GTTTGTGTATATTTACCTTACATTTATCTCC*-3′ to amplify the *Pfs47* gene fragment of 3,319 bp. HotStar HiFidelity Polymerase (Qiagen, NL) and long PCR amplification program was used; 5 min at 94 °C and then 30 s at 94 °C, 60 s at 48 °C, 10 min at 62 °C for 30 cycles and finally 15 min at 68 °C. Amplification products were then analysed on 1% agarose gel and purified using QIAquick Gel Extraction Kit (Qiagen, NL) or AMPure PCR DNA purification kit (Agencourt, MA). Sequence analysis was performed in triplicate or quadruplicate between nucleotides 0–417 using primer *BVS202* rev 5′-*CCGTTTTACTATTATCACATCTAC*-3′ and between nucleotides 500–860 using primer *MWV325* fwd 5′-*GTAGATGTGATAATAGTAAAACGG*-3′ (based on data of other *P. falciparum* strains the *Pfs*47 locus between nucleotide 417–500 was shown to have no polymorphisms). Results were compared to available *P. falciparum Pfs*47 sequences (i.e. NF54, 7G8) using Jalview, SegScape and Vector NTI (Invitrogen, UK).

### Statistical analysis

Statistical analyses were performed in STATA version 12 (Statacorp, College Station, Texas, US). The effect of *TEP1* knockdown was determined by comparing oocyst burden after *dsTEP* injection and *dsLacZ* injection. For this, a negative binomial regression model was fitted to the oocyst count data per parasite isolate and per mosquito strain using random-effects and conditional fixed effects overdispersion models (xtnbreg command in Stata), combining data from all experiments and adjusting for correlations between observations from the same experiment. A potential effect of injection on oocyst development was tested by comparing oocyst burden after *dsLacZ* with that in noninjected mosquitoes, using the same approach.

## Additional Information

**How to cite this article**: Eldering, M. *et al.* Variation in susceptibility of African *Plasmodium falciparum* malaria parasites to TEP1 mediated killing in *Anopheles gambiae* mosquitoes. *Sci. Rep.* **6**, 20440; doi: 10.1038/srep20440 (2016).

## Supplementary Material

Supplementary Information

## Figures and Tables

**Table 1:** The effect of *TEP1* knockdown on oocyst burden of three African *P. falciparum* isolates in susceptible (Ngousso) and resistant (L3–5) strains of *A. gambiae*.

		TEP1 knockdown effect	
*A. gambiae* Ngousso	*P. falciparum* isolate	Oocyst burden incidence rate-ratio (95% CI)	p-value
	NF54	1.11 (0.91–1.34)	0.30
	NF166	1.16 (0.90–1.49)	0.25
	NF165	1.70 (1.32–2.19)	<0.001
*A. gambiae* L3–5			
	NF54	1.22 (0.90–1.66)	0.20
	NF166	1.77 (1.31–2.40)	<0.001
	NF165	3.56 (2.36–5.39)	<0.001

The effect of *TEP1* knockdown was determined by comparing the oocyst burden in mosquitoes after *dsTEP1* injection compared to *dsLacZ* injection.
