# Supplementary material for: Variation in susceptibility of African Plasmodium falciparum malaria parasites to TEP1 mediated killing in Anopheles gambiae mosquitoes
Source: Sci Rep. 2016 Feb 10;6:20440. doi: 10.1038/srep20440 (PMC4748223; doi:10.1038/srep20440)
Supplement: Supplementary Information [file srep20440-s1.pdf]

Supplementary information

Variation in susceptibility of African *Plasmodium falciparum* malaria parasites to TEP1 mediated killing in *Anopheles gambiae* mosquitoes

Maarten Eldering[1,2], Isabelle Morlais[3], Geert-Jan van Gemert[1], Marga van de Vegte-Bolmer[1], Wouter Graumans[1], Rianne Siebelink-Stoter[1], Martijn Vos[1], Luc Abate[3], Will Roeffen[1], Teun Bousema[1,4*], Elena A. Levashina[2*], Robert W. Sauerwein[1*]

[1] Department of Medical Microbiology, Radboud University Medical Centre, Nijmegen, The Netherlands

[2] Vector Biology Unit, Max Planck Institute for Infection Biology, Berlin, Germany

[3] UMR MIVEGEC UM1-CNRS 5290-IRD 224, Institut de Recherche pour le Développement, Montpellier cedex, France

[4] Department of Immunology and Infection, London School of Hygiene and Tropical Medicine, London, United Kingdom

* Corresponding authors

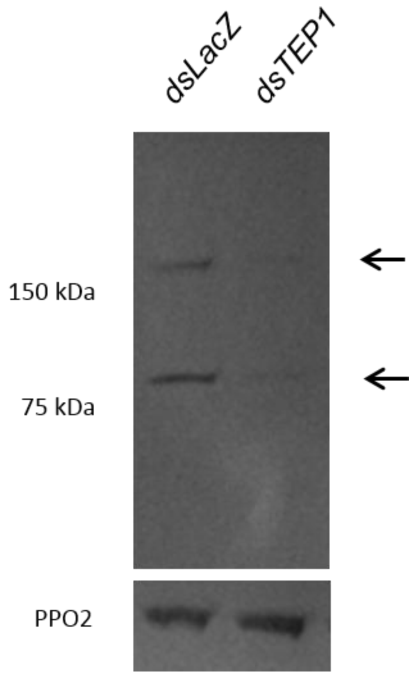

S1 Fig. Confirmation of *TEP1* knockdown in adult S female mosquitoes. The full-length and processed forms (arrows) of TEP1 were detected by immunoblotting in the hemolymph of control injected mosquitoes (*dsLacZ*), and almost complete absent in the hemolymph of mosquitoes injected with *dsTEP1*. Hemolymph PPO2 served as loading control.

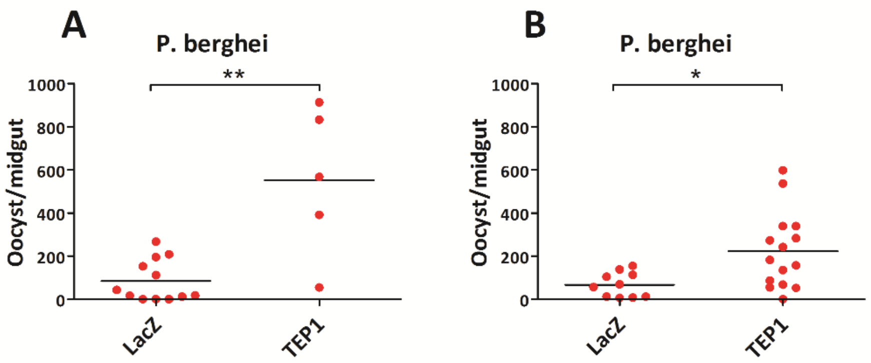

S2 Fig. Effect of *TEP1* silencing in *A. gambiae* S mosquitoes on *P. berghei* infection. Female mosquitoes were injected with *dsLacZ* (control) or *dsTEP1* 4 days prior to feeding on a *P. berghei* infected mouse. Oocysts were visualized on day 7-8 post infection (p.i.) by mercurochrome staining of midguts and counted by microscopy. (*A*) Comparison of *P. berghei* live oocysts numbers between control injected and *dsTEP1* injected group for experiment 1. (*B*) Comparison of *P. berghei* live oocysts numbers between control injected and *dsTEP1* injected group for experiment 2. Significance for difference between groups are indicated:*p<0.05, **p<0.005 (negative binomial regression model).

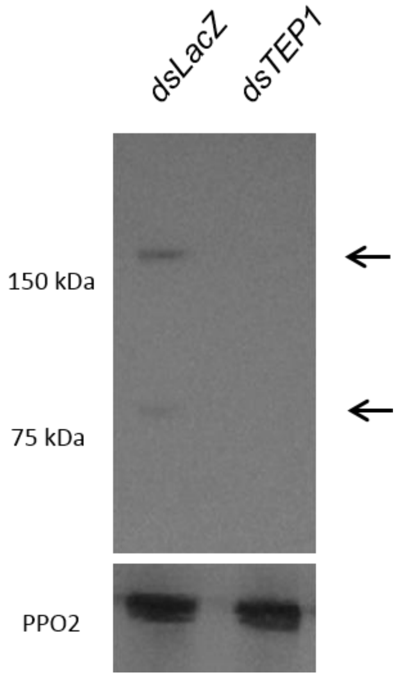

S3 Fig. Confirmation of *TEP1* knockdown in adult R female mosquitoes. The full-length and processed forms (arrows) of TEP1 were detected by immunoblotting in the hemolymph of control injected mosquitoes (*dsLacZ*), and were absent in the hemolymph of mosquitoes injected with *dsTEP1.* Hemolymph PPO2 served as loading control.

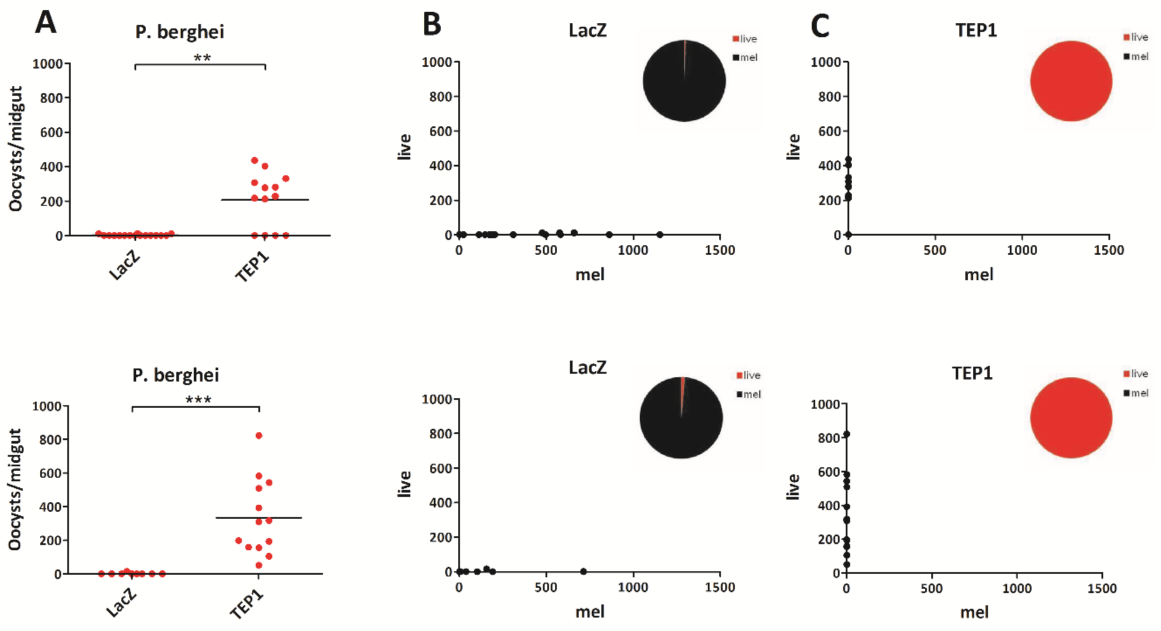

S4 Fig. Effect of *TEP1* silencing in *A. gambiae* R mosquitoes on *P. berghei* infection. Female mosquitoes were injected with *dsLacZ* (control) or *dsTEP1* 4 days prior to feeding on a *P. berghei* infected mouse. Oocysts were visualized on day 7-8 p.i. by mercurochrome staining of midguts and counted by microscopy. (*A*) Comparison of *P. berghei* live oocysts numbers between control injected and *dsTEP1* injected group. (*B*) Number of live and melanized parasites on individual midguts in the control group. Proportion of live/melanized is depicted in the pie chart. (*C*) Number of live and melanized oocysts on individual midguts after *TEP1* silencing. Proportion of live/melanized is depicted in the pie chart. Significance for difference between groups are indicated: **p<0.005, ***p<0.001 (negative binomial regression model).

		Injection effect	
A. gambiae Ngousso		Oocyst burden incidence rate-ratio (95% CI)	p-value
	NF54	0.81 (0.66-0.98)	0.033
	NF166	0.45 (0.35-0.58)	<0.001
	NF165	0.29 (0.23-0.36)	<0.001
A. gambiae L3-5			
	NF54	0.64 (0.45-0.91)	0.014
	NF166	0.97 (0.69-1.37)	0.86
	NF165	0.62 (0.40-0.96)	0.031

S1 Table. The wounding effect of injection on oocyst burden for three African *P. falciparum* isolates and two African *A. gambiae* strains. The effect of injection was determined by comparing the oocyst burden in mosquitoes after *dsLacZ* injection compared to mosquitoes that were not injected.

Region	Group	Isolate	532	581	657	671	707	718	725	740	742	814	817
Sub Saharan Africa	Laboratory isolates	3D7	A	C	G	T	C	C	C	T	A	A	T
		**NF54#**	.	.	.	.	.	.	.	.	.	.	.
		**NF165#***	.	A	.	.	.	.	.	.	.	.	.
		**NF166#**	.	A	A	.	.	.	.	.	T	T	.
	Cameroon	**ME5#***	.	A	A	.	.	.	.	.	T	T	.
		**MJ5#***	.	A	A	.	.	.	.	.	T	T	.
		**MR7#***	.	A	.	.	.	.	.	.	.	.	.
		**GJ5***	.	A	.	.	.	.	.	.	.	.	.
		**NES#***	.	A	.	.	.	.	.	.	.	.	.
		**SIL***	.	A	.	.	.	.	.	.	/T	.	.
		**LN5#**	.	A	.	.	.	.	.	.	.	.	.
		**BA8**	.	A	A	.	.	.	.	.	.	T	.
		**MA7**	.	/A	.	.	.	.	.	.	.	.	.
		**MF5**	.	A	.	.	.	.	.	.	/T	.	.
		**EVA**	.	A	.	.	.	.	.	.	/T	.	.
		**EA8**	.	A	.	.	.	.	.	.	/T	.	.
		**MI**	.	A	.	.	.	.	.	.	.	.	.
		**NH8**	.	A	/A	.	.	.	.	.	T	.	.
		**OJ1**	.	A	.	.	.	.	.	.	.	.	.
	Mali	Mali	.	A	.	.	.	.	.	.	.	.	.
	Tanzania	Tanzania	.	A	.	.	.	.	.	.	.	.	.
	Uganda	Uganda	.	A	.	.	.	.	.	.	T	T	A
Latin America	Laboratory isolates	7G8*	.	A	.	.	T	.	T	C	T	.	.
		Santa L	G	A	.	.	T	.	T	.	T	.	.
Philippines		FCH	G	A	.	.	T	.	T	.	T	.	.
South-East Asia	Malaysia	Malaysia	.	A	.	.	.	.	.	.	T	T	A
	Vietnam	UGT5	.	A	.	.	.	.	.	.	T	.	.
			.	A	.	A	T	A	.	.	T	T	.
	Cambodia	NF135	.	A	.	A	T	A	.	.	T	T	.

S2 Table. *Pfs47* polymorphic sites in laboratory and field *P. falciparum* isolates. *Pfs47* fragment from the laboratory strains NF54 (unknown), NF166 (Malawi) and NF165 (Guinea) of African origin, 15 field isolates used for experimental infections in Cameroon (Nsango et al, 2012) and 11 isolates from Latin America laboratory strains, Philippines and South-East Asia, were PCR amplified and sequenced. The isolates with known phenotypes for susceptibility to TEP1 and wounding are shown in bold (# - susceptible to wounding, * - susceptible to TEP1). Dots indicate nucleotides identical to the sequence of *P. falciparum* 3D7/NF54 strains. Nucleotide numbers for each sequence are given vertically at the top in relation to the respective PlasmoDB 3D7 sequence.
